# A first generation BAC-based physical map of the rainbow trout genome

**DOI:** 10.1186/1471-2164-10-462

**Published:** 2009-10-08

**Authors:** Yniv Palti, Ming-Cheng Luo, Yuqin Hu, Carine Genet, Frank M You, Roger L Vallejo, Gary H Thorgaard, Paul A Wheeler, Caird E Rexroad

**Affiliations:** 1 National Center for Cool and Cold Water Aquaculture, ARS-USDA, 11861 Leetwon Road, Kearneysville, WV 25430, USA; 2 Department of Plant Sciences, University of California, One Shields Ave., Davis, CA 95616, USA; 3 INRA, UMR1313, Genetique Animale et Biologie Integrative, Domaine de Vilvert, 78352 Jouy en Josas Cedex, France; 4 School of Biological Sciences and Center for Reproductive Biology, Washington State University, Pullman WA 99164-4236, USA

## Abstract

**Background:**

Rainbow trout (*Oncorhynchus mykiss*) are the most-widely cultivated cold freshwater fish in the world and an important model species for many research areas. Coupling great interest in this species as a research model with the need for genetic improvement of aquaculture production efficiency traits justifies the continued development of genomics research resources. Many quantitative trait loci (QTL) have been identified for production and life-history traits in rainbow trout. A bacterial artificial chromosome (BAC) physical map is needed to facilitate fine mapping of QTL and the selection of positional candidate genes for incorporation in marker-assisted selection (MAS) for improving rainbow trout aquaculture production. This resource will also facilitate efforts to obtain and assemble a whole-genome reference sequence for this species.

**Results:**

The physical map was constructed from DNA fingerprinting of 192,096 BAC clones using the 4-color high-information content fingerprinting (HICF) method. The clones were assembled into physical map contigs using the finger-printing contig (FPC) program. The map is composed of 4,173 contigs and 9,379 singletons. The total number of unique fingerprinting fragments (consensus bands) in contigs is 1,185,157, which corresponds to an estimated physical length of 2.0 Gb. The map assembly was validated by 1) comparison with probe hybridization results and agarose gel fingerprinting contigs; and 2) anchoring large contigs to the microsatellite-based genetic linkage map.

**Conclusion:**

The production and validation of the first BAC physical map of the rainbow trout genome is described in this paper. We are currently integrating this map with the NCCCWA genetic map using more than 200 microsatellites isolated from BAC end sequences and by identifying BACs that harbor more than 300 previously mapped markers. The availability of an integrated physical and genetic map will enable detailed comparative genome analyses, fine mapping of QTL, positional cloning, selection of positional candidate genes for economically important traits and the incorporation of MAS into rainbow trout breeding programs.

## Background

Rainbow trout (*Oncorhynchus mykiss*) are the most-widely cultivated cold freshwater fish in the world and are considered by many to be the "aquatic lab-rat". Interests in the utilization of rainbow trout as a model species for genome-related research activities focusing on carcinogenesis, toxicology, comparative immunology, disease ecology, physiology, transgenics, evolutionary genetics, and nutrition have been well documented [1]. Coupling great interest in this species as a research model with the need for genetic improvement for aquaculture justifies the continued development of genome resources facilitating selective breeding.

Genome size estimates derived from molecular weight of DNA per cell for rainbow trout and other salmonids vary from 2.4 to 3.0 × 10^9 ^bp [2,3]. As with most salmonids, rainbow trout experienced a recent genome duplication event resulting in a semi-tetraploid state [4]. Our physical mapping experience with BACs from the Swanson library has demonstrated that duplicated loci can be detected by DNA fingerprinting [5]. Additionally, BACs that represent one of two duplicated loci were shown by fluorescent *In-situ *hybridization (FISH) to distinctly hybridize to a specific chromosome pair [6]. Therefore, it is likely that the vast majority of the duplicated loci contain enough sequence variation to allow correct assembly of a physical map using BAC DNA fingerprinting.

Current genomic resources available for rainbow trout research include multiple bacterial artificial chromosome (BAC) libraries [5,7]; doubled haploid (DH) clonal lines [8-11]; genetic maps [3,12-15]; a large EST database [16,17]; and DNA microarrays [18,19].

Seven rainbow trout BAC libraries were constructed to date. Two libraries constructed in Japan [7] contain average insert sizes of 58 kb and 110 kb, and provide haploid genome coverages of 6.7 fold and 5.3 fold, respectively. However, they have not been arrayed in plates and library screening tools are not available. One BAC library from the Swanson male homozygous line and one from the OSU female homozygous line were commercially constructed by Amplicon Express Inc. in 2002. Both libraries were prepared from partial digestions with HindIII. The OSU BAC library has 96,768 clones with an average insert size of 110 kb (4.5× coverage). The Swanson BAC library has 184,704 clones with an average insert size of 130 kb (10× coverage). HindIII BAC DNA fingerprinting for local physical mapping of 27 Type-I markers in the Swanson library demonstrated the library's utility for identifying duplicated loci and confirmed its 10× coverage [5]. Both libraries have been used for genomic sequencing and genetic mapping of loci of interest [20-27]. An additional 5× genome coverage Swanson DH YY male library (92,160 clones) was constructed at the Children's Hospital Oakland Research Institute (CHORI-220; ) in 2005 using EcoRI partial digestion of genomic DNA with an estimated average insert size of 159 kb. An additional two new libraries were prepared by Amplicon Express in 2008. The two new 5× genome coverage Swanson DH YY male libraries (110,592 clones each) were prepared using BamHI and EcoRI partial genomic digestion to complement the 10X HindIII Swanson library and have estimated insert sizes of 140 kb. The four Swanson DH YY male libraries described above were prepared using genomic DNA from the same Swanson doubled-haploid clonal line that is propagated and maintained at the lab of Gary Thorgaard in Washington State University.

Two genetic maps with improved marker densities were recently developed for rainbow trout by INRA [12] and the NCCCWA [15]. The INRA map is based on a panel of two DH gynogenetic lines. It has more than 900 microsatellites over 31 linkage groups and a total length of 2,750 cM (average resolution of 3 cM). The NCCCWA map is based on a panel of five families that represent the starting genetic material of the NCCCWA selective breeding program. It has 1,124 microsatellite loci over 29 linkage groups and a total length of 2,927 cM (average resolution of 2.6 cM).

The rainbow trout haploid karyotype is composed of 52 chromosome arms, but chromosome numbers can vary among rainbow trout populations in concordance with their native geographic distribution [28]. Therefore, anchoring the genetic linkage groups to the physical chromosome arms was a crucial task accomplished by Phillips et al. [6] using BACs as FISH probes. The range of the haploid chromosome number (N) is between 29 and 32 [28]. The karyotype of the Swanson DH line is composed of 2N = 58 [29]. The offspring of "hybrids" between strains with different chromosome number are viable and they can be used for genetic mapping as two uni-armed (acrocentric) chromosomes from the parent with 2N = 60 will align with a di-armed (metacentric) chromosome from the parent with 2N = 58. A comparative cytogenetic map of the rainbow trout and Atlantic salmon using FISH with BACs that harbor Type-I markers and microsatellites is being developed in a coordinated effort [30]. This cytogenetic map and the comparative genetic map of Danzmann et al. [31] provide a frame-work for future high resolution trout-salmon comparative genome maps.

Qualitative/quantitative trait loci (QTL) mapping experiments in rainbow trout have been very successful because of their high fecundity, external fertilization, and ease of gamete handling and manipulation. Many QTL have been identified for production and life-history traits including resistance to the parasite *C. shasta *[32], resistance to IHNV [33,34] and to IPNV [35], Killer cell-like activity [36], upper thermal tolerance [37,38], embryonic developmental rate [8,39,40], spawning time [41,42], confinement stress response [43] and smoltification [44,45]. The availability of a BAC physical map integrated with the genetic map will facilitate fine mapping of QTL, the selection of positional candidate genes and the incorporation of marker-assisted selection (MAS) into rainbow trout breeding programs. A major shortcoming of QTL studies is that they are limited to the variation present in a limited number of families and typically do not detect loci with small effect. This can be overcome by whole genome association studies and other approaches that capture effects of most QTL that contribute to the population-wide variation in a trait such as genomic selection. Recently we demonstrated the feasibility of low resolution LD association studies in rainbow trout [46]. In the absence of whole genome sequence assembly, the robust integrated physical and genetic map that we aim to construct will provide better resolution than the current genetic maps for ordering of genetic markers and estimating physical distances between markers, thus facilitating whole genome association studies rainbow trout.

Several BAC-based physical maps were constructed in recent years for economically important aquaculture species including tilapia [47], Atlantic salmon [2] and catfish [48,49]. Here we report the construction of the first physical map of the rainbow trout genome using a 10× genome coverage BAC library derived from the Swanson DH clonal line.

## Results and Discussion

### BAC Fingerprinting and contigs assembly

**Figure 1:**
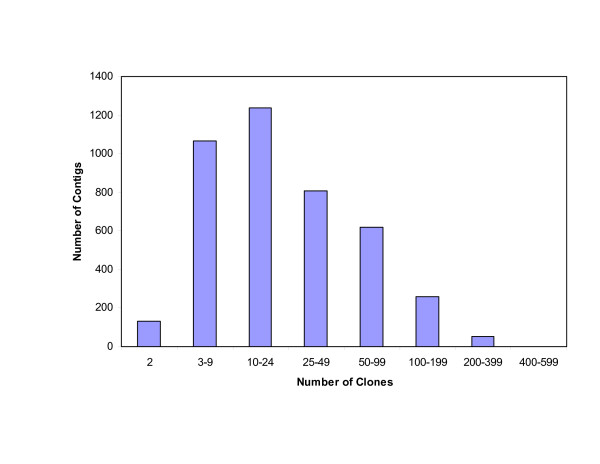
**BAC clones distribution in contigs**.

We used the 4-color High-Information-Content Fingerprinting (HICF) SNaPshot method of Luo et al. [50] to fingerprint all the clones from the 10X HindIII BAC library (184,704 clones) and 7,392 clones from the CHORI-220 5X EcoRI library. After editing with FPMiner software (BioinforSoft, Beaverton, OR) 82% BAC fingerprints from the 10X library and 50% from the CHORI-220 library were assembled into physical contigs using FPC software  with a tolerance of 0.5 bp and an initial cutoff of 1 E-70 (1 × 10^-70^), followed by DQer and several rounds of end-to-end merging and single-to-end merging at progressively lower cutoff stringencies. The current version of the map is composed of 154,439 clones of which 145,060 are assembled into 4,173 contigs and 9,379 remained singletons (Table 1). The average number of BACs per contig is 34.76, and the distribution of the number of BACs per contig is shown in Figure 1. The average number of fingerprinting fragments per BAC is 76.4, and the average insert size for this library is 130 kb [5]. Therefore, each fragment is estimated on average to represent 1.7 kb of genome DNA. The total number of unique fingerprinting fragments (consensus bands) in contigs is 1,185,157, which corresponds to an estimated physical length of 2.0 Gb (75% - 80% of the rainbow trout genome). The average number of consensus bands (CB) per contig is 284, and the estimated contig size is 482 kb. The number of contigs in this assembly is similar to the first generation Atlantic salmon physical map, which resulted in 4,354 contigs and 37,285 singletons with an average contig length of 590 kb from fingerprinting of a 12.5X library [2]. The rainbow trout physical map can be searched and viewed online via WebFPC: 

**Table 1:** BAC fingerprinting and FPC assembly statistics

Number of clones fingerprinted	192,096	~10.4× genome coverage
Used in FPC assembly	154,439	~8.3× genome coverage
Average insert size (Kb)	130	
Average number of fragments per clone	76.4	
Estimated average fragment size (Kb)	1.7	
Number of clones in contigs	145,060	~7.8× genome coverage
Number of contigs	4,173	
Average number of clones per contig	34.76	
Average contig size in consensus bands	284	
Estimated average contig size (Kb)	482	
Number of Q contigs	811	19.4%
Number of Q clones	1,986	1.4%
Number of CB in contigs	1,185,157	
Estimate total length in contigs (Kb)	2,000	70% - 80% genome size

### Validation of contigs

The physical map assembly was validated by: 1) comparing contigs assembly to the probe hybridization results and agarose gel fingerprinting contigs of Palti et al. [5]; and 2) anchoring large contigs to the microsatellite-based genetic linkage map of Rexroad et al. [15]. In the first approach we evaluated the contig assignments of 236 clones that were positive by probe hybridization to 27 type I markers (Table 2). Most of the clones (189) that were positive for a single probe and were also assembled into a single contig by Palti et al. [5] also clustered inside a single contig in this physical map, confirming the reliability of this assembly. Only one marker (*fabp7b*) was truly split into two contigs in the physical map where five clones clustered in contig 2908 and four clustered in contig 1658. The other 10 clones that did not cluster in the major contig of clones positive to each marker did not cluster with other positive clones either, likely representing the fraction of mis-assembled clones. An additional 33 clones (14%) that were positive to the markers of Palti et al. failed our fingerprinting editing criteria and were excluded from the current FPC assembly. Overall, the current assembly of 93% of the clones was in agreement with our previous work and only 10 clones (5%) were likely mis-assembled.

**Table 2:** Assembly of BACs positive to gene probes and the agarose HindIII fingerprinting of Palti et al. [5].

**Gene**	**Major Contig**	**No. of Clones**	**Other Contigs**^**a**^	**Failed**^**b**^
1. a1-mg-1	4410	5		1
2. CXC-R4	1145	8	1 (348)	1
3. DAB	Singleton	1		
4. fabp7b	2908	5	4 (1658), 1 (566)	2
5. GH2-1	228	10		2
6. GH2-2	560	4		
7. GTPBP-Gi-1	2715	5		1
8. GTPBP-Gi-2^c^	4594	7	1 (247)	
9. GTPBP-Gi-3^c^	4594	3		1
10. GTPBP-Gi-4	608	5		
11. Hep-1	336	17	1 (9737)	4
12. Hep-2	12	7		
13. HSZFP238-a	1304	16		2
14. HSZFP238-b	3300	2		
15. ID1B	1492	16		2
16. ID1C-1	2759	3		2
17. Irp-1A-1	2251	9		2
18. Irp-1A-2	1106	7	1 (761)	3
19. MHCIa-1	3093	8	1 (450), 1 (1607), 1 (1937)	3
20. MHCIa-2	84	9	1 (1304)	1
21 MHCIa-3	959	8	1 (3816)	
22. NPY-1	5683	4		
23. NPY-2	3205	2		
24. RP-S16-a	2361	5		2
25. RP-S16-b	1657	3		
26. SCAR163	3249	9		1
27. TAP1	260	11		3

Total:		189 (93%)	14 (7%)	33 (14%)

^**a **^The contig numbers are in parenthesis.

^**b **^Clones that were not fingerprinted successfully and did not pass the editing step of the analysis.

^**c **^GTPBP-Gi-2 and GTPBP-Gi-3 appear tightly linked on contig 4594, but the respective positive BACs do not overlap. This may be caused by local tandem duplication of this locus.

**Figure 2:**
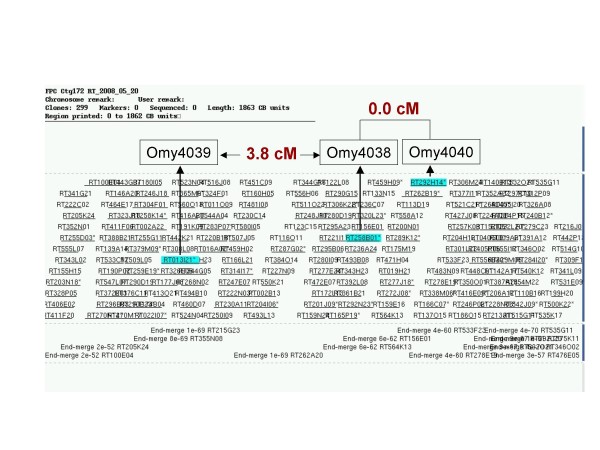
**Example of a rainbow trout contig anchored to a genetic linkage group using microsatellites isolated from BAC end sequences**. The contig shown is number 172 containing 299 clones. The 3 markers were mapped to Chromosome 6. Genetic distances between markers were calculated from a two-point linkage analysis. The clones from which the markers were isolated (Table 3) are "hidden" within the highlighted clones. Clone groups with more then 90% DNA fingerprints overlap are represented by the largest BAC in the group, which was not the actual BAC from which the microsatellites shown here were isolated.

In the second validation approach, 11 of the largest contigs were anchored to the genetic linkage map using 25 microsatellite markers isolated from BAC end sequences (BES) and reported here for the first time, and three microsatellites that were previously isolated from other clones in the contigs (Tables 3 and 4). Two to four markers were developed per contig from clones that were distal to each other on the contig as illustrated in Figure 2. All of the 28 markers were placed on the rainbow trout genetic map by two-point linkage analysis. Markers from 9 of the 11 contigs displayed close genetic linkage of 0 cM - 10 cM. The other two contigs (138 and 450) were likely mis-joined as for each contig two of the three markers were closely linked and the third marker was mapped to another linkage group (Table 4). Those two were also the largest contigs with 334 and 431 clones and estimated length of 3.7 Mb and 4.6 Mb, respectively. The ratio of physical to genetic linkage distances varied among the contigs we sampled, which is similar to other vertebrate genomes [48,51]. We will be able to better investigate this relationship in the rainbow trout genome when we will have a comprehensive and robust integration between the physical and genetic maps. In terms of number of contigs, 9 of 11 (82%) are in agreement with the genetic map. In terms of genome coverage in number of markers, 24 of 26 (92%) are in agreement between the physical and genetic maps. This 8%-18% error rate is higher then the 5% estimated for the catfish physical map of Quiniou et al. [48] or the 4% rate detected in the 3-color HICF physical map of the maize genome [52]. However, the whole assembly error rate for this trout physical map is likely lower than the estimate of 18% or even 8% as this validation was heavily skewed toward the largest contigs, and indeed the two mis-joined contigs were also the largest contigs.

**Table 3:** Genetic linkage mapping of microsatellites isolated from BAC end sequences.

**Marker**	**Clone**	**Contig**	**Chr.**	**Forward/Reverse Primers**	**GenBank**
OMY4002	170E02	58	14	AGGTTATTTCCATTTCCCGC/GAGGAGTCCCAGAGGAAAGG	GF100674
OMY4003	378C10	58	14	GACTTCTGCTCTGTCGGTCC/GACAGGTAGCCAAAACTCCG	GF100675
OMY4005	116G20	260	2	TCATAAGTCATATGGTGACTATCATTT/GCAAATGCATTGACATCTCG	GF100676
OMY4006	162L19	260	2	GATACACCCCTGCTGTTCGT/ACCCACCAAGCCACTCTCT	GF100677
OMY4007	146D09	260	2	AACGCATAGGAGGGAGGATT/AAAATATTGTGGCCAGCAGC	GF100678
OMY4008	198M04	84	18	ATGCTTTTGCAATTTCCTGG/ATGTTCATGCTGACCGACTG	GF100698
OMY4009	227H04	791	22	CGCTGGAATGTTTTCATCTG/ATTTCACAAATGGCCAGGAG	GF100679
OMY4010	383M11	930	11	TGATCATGGCACCCATACTG/TCACCCTGGTGGCCTACTAC	GF100680
OMY4011	154C16	930	11	CAGTATGTCCTGTGAGGCCC/TCCACTTTAAGGGCATTTGG	GF100681
OMY4012	194O09	100	27	AGCAAGCTCAATGAAGCACA/GAGCCCAGAGGTGAGATCAG	GF100682
OMY4013	251I01	100	27	AGCGGACTGGGCTGTAATAA/ATGGACCAACTGAGCCTGAC	GF100683
OMY4015	318K03	138	27	GTGGGCATTTTTGCTGACTT/CCGTTGATACATTTTGGCAG	GF100684
OMY4017	300B08	138	1	TCATCTTCGACAGCATGGAG/GAAGGCCAAAGAAGCATGAA	GF100685
OMY4018	207I23	138	1	CCTGTTTTGAAAATGGGACC/ACCAACCGCCATAGTAGCAG	GF100686
OMY4020	377G20	168	10	TGTCCCTCAAAGTGCTACCC/CAGATGTGGGAACTTGAGCA	GF100687
OMY4022	207O04	168	10	ACAAAGACCACAACGGCATT/TTGGCATTTACATATGTCCCC	GF100688
OMY4023	241D02	336	3	ATCTCCAAGCCCTGAGGAAT/TTTTTGGTCCCCACAAGAAT	GF100689
OMY4024	203O06	336	3	GAGCCAGTAATTCATTCGCC/GCAGGACAATCGTTTTAGGG	GF100690
OMY4025	278E22	336	3	ATGACCCTGACGGGATGTTA/AGCTCCACACACAACACAGC	GF100691
OMY4026	178K11	450	22	GTCGCAAAAGGCACTAAAGG/TGTGGCAGGTGCTGTTAGAG	GF100692
OMY4027	204J15	450	22	ATGCCAAAGAAATGGACAGG/TGGCCTCCCTTGTCATTAAA	GF100693
OMY4028	220L14	450	1	TCCCAGTGGATGGGACTTAG/GTGGGTGTCACATGTGTGGT	GF100694
OMY4038	218N06	172	6	GGGGAAATTCAACCCACTTT/ATGGCGAATTGGCTAGACTG	GF100695
OMY4039	275M02	172	6	ACTCTCCCCTGTCCTCCATT/CTAGTATCGACCCCTGCGAA	GF100696
OMY4040	386B11	172	6	TGAAGGGGGCTGATTAGTTG/ACAGCGTTCCATAGCGAGAT	GF100697

All clones were from the 10X HindIII YY male Swanson doubled haploid BAC library (RT).

**Table 4:** Validation of physical map assembly by linkage mapping of microsatellites isolated from clones that were part of 11 of the largest contigs in the rainbow trout physical map.

**Contig**	**No. of Clones (Q)**^**a**^	**Contig Length (Kb)**	**No. of Markers**	**r**^**b**^	**LOD**	**Chr.**
58	174 (3)	1,938	2	0.000	29.2	1
84^c^	190 (3)	2,300	2	0.014	19.1	18
100	313 (2)	3,300	2	0.101	20.6	27
168	306 (3)	2,934	2	0.009	28.7	10
172	299 (6)	3,167	3	0.040	8.1	6
260^c^	224 (1)	2,692	4	0.039	31.3	2
336	136 (0)	1,722	3	0.005	46.3	3
791^c^	124 (0)	1,722	2	0.000	25.2	22
930	112 (1)	1,394	2	0.006	49.7	11

Mis-joined Contigs						
138a	431 (4)	4,590	2	0.043	16.0	1
138b	431 (4)	4,590	1	N/A	32.0	27
450a	334 (4)	3,709	2	0.006	38.6	22
450b	334 (4)	3,709	1	N/A	39.4	1

^**a **^Number of Q-clones in parenthesis. Clones that were assigned to a contig but may be false positives (e.g. chimerical clones) are marked by FPC as Q-clones.

^**b **^Two point linkage recombination between markers from the two most distant clones in the contig.

^**c **^Additional markers that were previously isolated from other BACs in the contigs were linked to the new BAC end sequence microsatellites by two point linkage analysis and included in this table. Contig 84: OMM3090/MHCI-A [27]; Contig260: OMM3079/TAP1 [23]; Contig 791: OMM3183/TLR8a (manuscript in preparation).

FPC identified 1,986 questionable (Q) clones in 811 contigs in this physical map (Table 1). Q-clones are the result of false overlaps between DNA fingerprinting patterns, which can be caused by the presence of chimerical clones in the BAC library, cross-contamination between neighboring wells, large repetitive regions of the genome or duplicated regions that are frequent in the trout genome. The occurrence of Q-clones in this assembly (1.4%) is lower than the 4%-11% reported for other HICF projects [48,49,52]. However, the fraction of contigs with Q-clones in this assembly (19.4%) is similar to the catfish physical map assembly of Xu et al. [49]. The initial high cutoff stringency and relatively deep genome coverage that we used likely contributed to the lower fraction of Q-clones in this assembly. The quality of this physical map was validated, but it could still benefit from better computational tools for identifying Q-clones. Clearly, the assembly of physical maps can be significantly improved by identifying the specific Q-clones in each contig, which in turn will enable evaluation of their location within the contig and relationship to the neighboring clones. As a proof of concept, the computational approach for improving physical maps assembly that is currently being developed by Frenkel et al. [53] was tested on the two large mis-joined contigs that we identified in this assembly and correctly identified the specific Q-clones causing the mis-joining of the contigs and how they should be split into smaller contigs that would also be in good agreement with the genetic map (data not shown). Contig 260 that was also analyzed by this approach was found to be an intact contig, which is also in agreement with our results (Table 4). Taken together the results of this analysis illustrate that better handling of Q-clones by the assembly software can dramatically improve physical maps.

## Conclusion

The production and validation of the first physical map of the rainbow trout genome is described in this paper. We are currently integrating this map with the genetic map using more than 200 microsatellites isolated from BAC end sequences and by identifying BACs that harbor more than 300 previously mapped markers. The availability of an integrated physical and genetic map will enable detailed comparative genome analyses, fine mapping of QTL, positional cloning, selection of positional candidate genes for economically important traits and the incorporation of MAS into rainbow trout breeding programs. A comprehensive integrated map can also provide a minimal tiling path for genome sequencing and a framework for whole genome sequence assembly.

## Methods

### BAC libraries and DNA fingerprinting

BAC clones were obtained from the DH Swanson YY male 10X HindIII library [5] and the CHORI-220 Swanson 5X EcoRI library . All the clones from the 10X HindIII BAC library (184,704 clones) and 7,392 clones from the CHORI-220 library, were fingerprinted. We used the 4-color HICF SNaPshot method of Luo et al. [50]. For each 384-well plate from the library four 96-well blocks were inoculated using a 96-pin replicator. For plate orientation and fingerprinting quality, wells E7 and H12 were replaced in each 96-well block with a control BAC clone. The cultures were grown on an orbital shaker at 37 C and 400 rpm for 22 hr. The BAC DNA was purified using the Qiagen R.E.A.L. 96 prep kit (Qiagen, Valencia, CA). Each BAC was simultaneously digested with four 6-bp recognizing restriction endonucleases generating 3' recessed ends and a 4-bp recognizing restriction endonuclease producing blunt ends. Each of the four recessed 3' ends was labeled with a different fluorescent dye using the SNaPshot kit (Applied Biosystems, Foster City, CA). Restriction fragments were sized with a capillary DNA analyzer ABI3730XL (Applied Biosystems) using an internal GeneScan-1200 LIZ v. 1 size standard. Fragment size-calling was performed with the GeneMapper v. 3.7 (Applied Biosystems). Outputs of size-calling files were automatically edited with the FPMiner program  using the program's default setting. This software package was used to distinguish peaks corresponding to restriction fragments from peaks generated by background noise in the profile of each BAC fingerprint and to remove vector restriction fragments from the profiles. The program also removed sub-standard profiles that could negatively affect contigs assembly. The files generated by FPMiner were used in the FPC contig assembly.

### Contigs assembly

Contigs were assembled from fragments within size range of 70-1,000 bp using FPC program version 8.5.3 [52,54]. FPC parameters were adjusted for the HICF method as previously described [48,50,52]. An initial assembly was performed with a tolerance of 0.5 bp and a Sulston score of 1 × 10^-70^. Contigs with more than 15% Q-clones were re-assembled by setting the DQer function to 15% and the "Step" value to 2. This was followed by several rounds of end-to-end merging and single-to-end merging at progressively lower cutoff stringencies (Table 5). The "Best of" function was set to 50 builds.

**Table 5:** FPC parameters used to assemble the physical map.

**Sulton Score**	**Step in the Assembly Build**	**Merge Function**
1.00E-70	Initial assembly	--
1.00E-70	Dqer, 15%, Step: 2	--
1.00E-65	Single-to-End	35
1.00E-55	Single-to-End	35
1.00E-68	End-to-End, 2	35
1.00E-60	End-to-End, 2	35
1.00E-50	End-to-End, 2	35
1.00E-45	Single-to-End	35
1.00E-38	Single-to-End	35
1.00E-40	End-to-End, 2	35
1.00E-35	End-to-End, 2	35
1.00E-50	End-to-End, 1	35
1.00E-40	singletons to contigs	--
1.00E-35	singletons to contigs	--

### BAC end sequencing and markers development

The 10X HindIII Rainbow trout BAC library [5] was used for BAC-end sequencing (BES). BAC culture was conducted using standard protocols and end sequencing with SP6 and T7 primers was done using standard Sanger technique. The raw, untrimmed files were processed by PHRED software [55]. The PHRED quality score cut-off value was set at 20 for the acquisition of Q20 values. The BESs were trimmed of vector sequences (pBeloBAC11 vector [56]) and filtered of *E. coli *sequences. Microsatellites and other simple sequence repeats (SSR) were analyzed using Tandem repeat Finder software [57]. We examined ten classes of SSRs by using a maximum period size of 10. BESs harboring at least 50 base pairs (bp) flanking sequences on either side of the microsatellites were selected for PCR primer design. Primers for BESs containing microsatellites were designed using Primer3 software [58]. The primer product size range was chosen between 150 and 450 nucleotides. The optimum size of primers was set to 20 nucleotides (range from 18 to 27 nucleotides) with an optimum melting temperature of 60.0°C (range from 57 to 63°C).

### Genotyping

The NCCCWA mapping panel of 5 families was genotyped with microsatellites as previously described [15]. A total of 25 microsatellite markers isolated from BAC end sequences (Table 3) and three microsatellites that were previously isolated from BAC clones [23,24,27] were genotyped using the tailed protocol of Boutin-Ganache *et al*. [59]. Primers were obtained from commercial sources (Alpha DNA, Montreal, Quebec, Canada). Three oligonucleotide primers were used in each DNA amplification reaction (Forward: 5' GAGTTTTCCCAGTCACGAC-primer sequence 3'; reverse: 5' GTTT-primer sequence 3'; fluorescent labeled primer with FAM: 5' GAGTTTTCCCAGTCACGAC 3'). Primers were optimized for amplification by varying annealing temperatures and MgCl2 concentrations. PCR reactions (12 μl total volume) included 50 ng DNA, 1.5-2.5 mM MgCl_2_, 2 pmol of forward primer, 6 pmol of reverse primer, 1 pmol of fluorescent labeled primer, 200 μM dNTPs, 1× manufacturer's reaction buffer, and 0.5 unit Taq Polymerase (ABI, Foster City, CA, USA). Amplifications were conducted in an MJ Research DNA Engine thermal cycler model PTC 200 (MJ Research, Waltham, MA) as follows: an initial denaturation at 95°C for 10 min, 30 cycles consisting of 94°C for 60 sec, annealing temperature for 45 sec, 72°C extension for 45 sec; followed by a final extension of 72°C for 10 min. PCR products were visualized on agarose gels after staining with ethidium bromide. Three μl of each PCR product was added to 20 μl of water, 1 μl of the diluted sample was added to 12.5 μl of loading mixture made up with 12 μl of HiDi formamide and 0.5 μl of Genscan 400 ROX internal size standard. Samples were denatured at 95°C for 5 min and kept on ice until loading on an ABI 3730 DNA Analyzer (ABI, Foster City, CA, USA). Output files were analyzed using GeneMapper version 3.7 (ABI, Foster City, CA, USA), formatted using Microsoft Excel and stored in a Microsoft Access database.

### Linkage analysis

The 28 microsatellites were placed on the rainbow trout genetic map by two-point linkage analysis as previously described [15,60]. Genotype data combined for both sexes were formatted using MAKEPED of the LINKAGE [61] program and checked for inconsistencies with Mendelian inheritance using PEDCHECK [62]. RECODE [63] and LNKTOCRI [64] were used to assemble the data into CRIMAP [65] format. Genotype data were added to that of Rexroad et al [15] and MULTIMAP [66] was used to conduct two-point linkage analyses to identify the closest markers from the published map having the highest LOD Scores.

## Authors' contributions

**YP **designed the study, conducted quality control analyses for the BAC fingerprints, collected microsatellite genotypes, validated contigs assembly and wrote the manuscript draft; **MCL **participated in the study design, supervised DNA extractions and BAC fingerprinting and assembled the physical map; **YH **conducted DNA extractions and BAC fingerprinting; **FMY **provided bioinformatics support for the fingerprints analyses and physical map assembly; **CG **obtained BAC end sequences, identified microsatellites and designed PCR primers; **RLV **participated in the study design and developed the genetic linkage analysis pipeline; **GHT **and **PAW **developed and maintained the Swanson DH line and provided blood from the clonal line as a source of genomic DNA for the BAC libraries; **CER **participated in the study design and conducted the genetic linkage analysis. All authors reviewed and contributed to the manuscript.
